# Prevention of Synaptic Alterations and Neurotoxic Effects of PAMAM Dendrimers by Surface Functionalization

**DOI:** 10.3390/nano8010007

**Published:** 2017-12-25

**Authors:** Felipe Vidal, Pilar Vásquez, Francisca R. Cayumán, Carola Díaz, Jorge Fuentealba, Luis G. Aguayo, Gonzalo E. Yévenes, Joel Alderete, Leonardo Guzmán

**Affiliations:** 1 Laboratory of Molecular Neurobiology, Department of Physiology, Faculty of Biological Sciences, University of Concepcion, Concepción 4070386, Chile; felipevidal@udec.cl (F.V.); pivasquez@udec.cl (P.V.); fcayuman@udec.cl (F.R.C.); 2 Laboratory of Biomaterials and Molecular Design, Department of Organic Chemistry, Faculty of Chemical Sciences, University of Concepcion, Concepción 4070386, Chile; caroladiaz@udec.cl (C.D.); jalderet@udec.cl (J.A.); 3 Department of Physiology, Faculty of Biological Sciences, University of Concepcion, Concepción 4070386, Chile; jorgefuentealba@udec.cl (J.F.); laguayo@udec.cl (L.G.A.); gyevenes@udec.cl (G.E.Y.)

**Keywords:** PAMAM dendrimers, nanocarriers, drug delivery, synaptic effects, neurotoxicity of nanomaterials, nanosafety, safe nanomaterial design

## Abstract

One of the most studied nanocarriers for drug delivery are polyamidoamine (PAMAM) dendrimers. However, the alterations produced by PAMAM dendrimers in neuronal function have not been thoroughly investigated, and important aspects such as effects on synaptic transmission remain unexplored. We focused on the neuronal activity disruption induced by dendrimers and the possibility to prevent these effects by surface chemical modifications. Therefore, we studied the effects of fourth generation PAMAM with unmodified positively charged surface (G4) in hippocampal neurons, and compared the results with dendrimers functionalized in 25% of their surface groups with folate (PFO_25_) and polyethylene glycol (PPEG_25_). G4 dendrimers significantly reduced cell viability at 1 µM, which was attenuated by both chemical modifications, PPEG_25_ being the less cytotoxic. Patch clamp recordings demonstrated that G4 induced a 7.5-fold increment in capacitive currents as a measure of membrane permeability. Moreover, treatment with this dendrimer increased intracellular Ca^2+^ by 8-fold with a complete disruption of transients pattern, having as consequence that G4 treatment increased the synaptic vesicle release and frequency of synaptic events by 2.4- and 3-fold, respectively. PFO_25_ and PPEG_25_ treatments did not alter membrane permeability, total Ca^2+^ intake, synaptic vesicle release or synaptic activity frequency. These results demonstrate that cationic G4 dendrimers have neurotoxic effects and induce alterations in normal synaptic activity, which are generated by the augmentation of membrane permeability and a subsequent intracellular Ca^2+^ increase. Interestingly, these toxic effects and synaptic alterations are prevented by the modification of 25% of PAMAM surface with either folate or polyethylene glycol.

## 1. Introduction

The improvement of the effectivity of pharmacological agents, such as small molecules, peptides and genes, is a permanent concern to the scientific community. These molecules need to overcome problems like insolubility, bioavailability, specific targeting, crossing biological barriers, and, in some cases, reaching intracellular targets [1,2,3]. One of the most remarkable strategies developed to overcome these difficulties is the use of drug delivery nanocarrier systems [4,5,6]. Among the nanomaterials used as nanocarriers, polyamidoamine (PAMAM) dendrimers appear as one of the most successfully used and studied [3,5,7]. These dendrimers are hyperbranched polymers organized from an ethylenediamine central core that gives way to expansive growing layers, known as generations, terminating in a surface of primary amines that are positively charged at physiological pH. This structure generates inner cavities, which are able to encapsulate small molecules and an easily modifiable surface that can covalently link pharmacological agents or ligands for specific targeting. Moreover, the terminal positive charges allow the electrostatic interaction with larger macromolecules such as nucleic acids [3,8,9].

A key aspect to study for the application of these polymers in effective therapies is their biocompatibility, i.e., to demonstrate the absence of toxic effects and the perturbation of physiological functions. Indeed, the cytotoxicity induced by surface positive charges of dendrimers, which increases with higher generations and concentration, has been previously described [10,11]. Studies in lipid bilayer models show that the cationic surface of PAMAM dendrimers interact with negatively charged biological membranes, leading to the disruption of their stability and the formation of nanoscale holes, which can end in cell lysis [12,13]. In addition, studies in different cell types demonstrate that oxidative stress and apoptosis are involved in cytotoxicity [14,15,16]. On the other hand, it has been described that dendrimers induce platelet aggregation depending on their generation and surface chemical groups [17]. To reduce these toxic effects, chemical modifications have been used linking different neutral or anionic molecules to the surface amine groups, resulting in an important enhancement of their biocompatibility [4,14,18,19].

Beyond these general aspects of toxicity or biocompatibility, it is highly relevant to know the specific interactions that these nanomaterials have with particular cell types and tissues. For applications in the central nervous system (CNS), different dendrimers have been used mainly to enhance the bioavailability, the specific targeting for this system and the ability to cross the blood-brain barrier of pharmacological agents [20,21,22]. It is also possible to mention specific studies related to internalization mechanisms of PAMAM dendrimers in neurons and glia cells [23,24,25]. In spite of the promising results obtained in in vitro and in vivo models for the treatment of glioma, cerebral palsy and neurodegenerative diseases [26,27,28], the biocompatibility and elimination of toxic effects of these polymers remain as a relevant challenge for their clinical application in CNS [3,29].

In regards to this aspect, it has been reported that PAMAM dendrimers induce apoptosis in cortical neurons [24] and autophagy that conduces to cell death in glioblastoma cell lines [30]. Additionally, evidence exists showing that G5 PAMAM dendrimers increase Na^+^ influx in pyramidal neurons, which could be mediated by membrane pore formation [31]. Furthermore, it has been shown that these molecules are capable of inducing a significant increase in intracellular Ca^2+^ leading to mitochondrial depolarization in pyramidal neurons and astrocytes [32].

Although the effects of dendrimers in cellular viability and membrane permeability have been reported in different studies, a deeper analysis of neurotoxic effects is necessary. In particular, the study of possible synaptic activity perturbation is a crucial aspect to be determined for the safe use of dendrimers in neurological applications. We hypothesize that unmodified dendrimers generate neurotoxic effects and alter the normal synapsis, which can be prevented by surface functionalization. Therefore, primary culture hippocampal neurons from E18 mice embryos were used as study model to determine the effects of fourth generation PAMAM dendrimers with a completely positively charged surface (G4), and dendrimers modified in 25% of their terminal groups with folate (PFO_25_) and polyethylene glycol (PPEG_25_). Cell viability and specific neuronal functions like membrane permeability, intracellular Ca^2+^ regulation, synaptic vesicle release and synaptic activity were analyzed.

## 2. Results and Discussion

### 2.1. PAMAM Dendrimers

**Figure 1:**
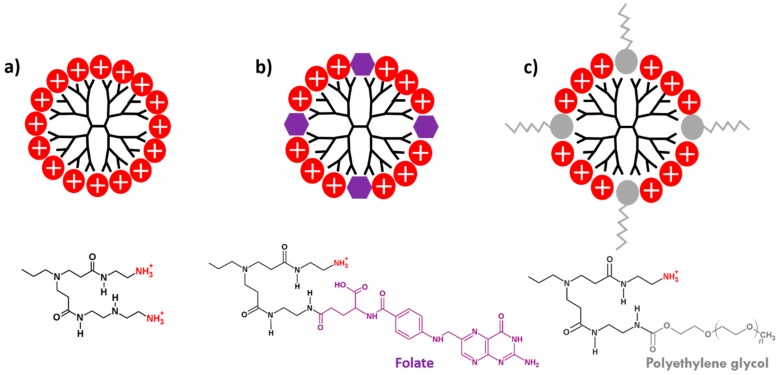
Schematic representation of studied dendrimers. (**a**) G4: Fourth generation PAMAM dendrimer with a completely positively charged surface (red); (**b**) PFO_25_: G4 functionalized in 25% of the surface with folate (purple); (**c**) PPEG_25_: G4 functionalized in 25% of the surface with polyethylene glycol (gray).

The use of drug nanocarrier systems to improve current and new therapeutic alternatives has been an important concern to pharmacological research [6,33]. Hyperbranched polymers like PAMAM dendrimers have been widely studied demonstrating a notable potential as drug delivery systems [3,5,7]. The surface properties of PAMAM dendrimers are a key feature that determine their biocompatibility [3,10,11]. Therefore, it is highly relevant to study their cytotoxic effects, neuronal activity alterations and the possibility to diminish those effects using different PAMAM-based dendrimers. For this reason, G4 fourth generation PAMAM dendrimer exposing its 64 positively charged amine surface groups was used to generate two new dendrimers functionalized with molecules of different sizes: (1) PFO_25_: G4 PAMAM, with 25% of its amine surface groups modified with folate (441.4 g/mol); and (2) PPEG_25_: G4 PAMAM, with 25% of its amine surface groups modified with polyethylene glycol (1900–2200 g/mol) (Figure 1). The effects of G4, PFO_25_ and PPEG_25_ in different parameters related to cell viability and normal neuronal activity were evaluated.

### 2.2. Cytotoxicity Evaluation

**Figure 2:**
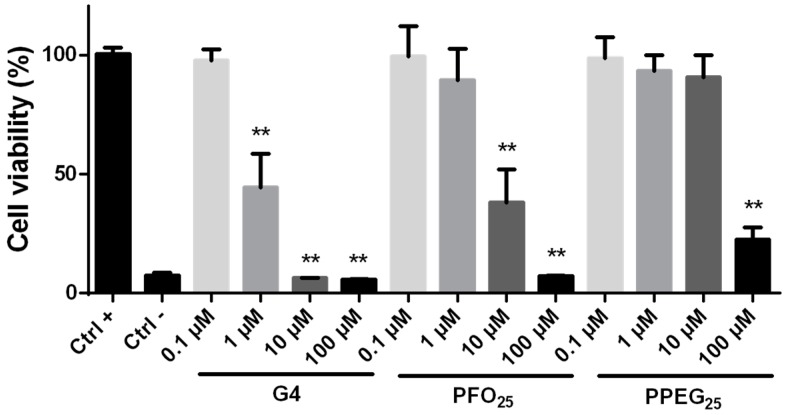
Cytotoxicity evaluation. Cell viability assay was performed incubating hippocampal neurons for 24 h with G4, PFO_25_ and PPEG_25_. Incubation only with culture media and Triton were used as positive and negative viability controls, respectively. Fluorescent emission of AlamarBlue reagent was measured and results are expressed as percentage of cell viability considering the fluorescence of positive control as 100% of viability. Significant differences between dendrimer treatments and positive control were analyzed (*n* = 6; ** *p* < 0.01).

To determine the cytotoxic effects that the different dendrimers (Figure 1) could cause in primary culture neurons, a cell viability assay (i.e., resazurin assay) was performed incubating cultures with increasing concentrations of G4, PFO_25_ and PPEG_25_ for 24 h. The results showed that G4 dendrimers induced a high level of cytotoxicity reducing neuron viability to 44.57 ± 13.93% at 1 µM. At higher concentrations of 10 and 100 µM, the cytotoxic level was comparable to the negative control reducing viability to 6.3 ± 0.10 and 5.7 ± 0.29%, respectively. In the case of PFO_25_, it was possible to observe a reduction in the toxic effect as compared to G4. The significant decrease in viability was observed starting at 10 µM with a value of 38.2 ± 13.87%, whereas a reduction similar to the negative control was obtained at 100 µM (7.1 ± 0.36%). On the other hand, PPEG_25_ exhibited a better biocompatibility performance demonstrating no toxicity at 0.1 to 10 μM. Significant toxicity was found only with the highest concentration used, with a reduction in viability to 22.45 ± 5.30% at 100 μM (Figure 2). These results corroborate the toxic properties of the high number of free positively charged amines and demonstrate that not only the number, but also the size and chemical properties of functional surface added molecules are important for the generation of more biocompatible nanocarriers.

Previous studies reported that G4 PAMAM dendrimers cause cytotoxicity in mHippoE-18 embryonic mouse hippocampal cells at concentration of 40 µM using the MTT cell viability assay and a 24 h incubation period [14]. In the present study, we showed that cytotoxic effects can be observed at concentrations as low as 1 µM. This difference could be explained due to the immortalized nature of the mHippoE-18 cell line, which would be more resistant to toxic agents than primary culture neurons. The significant reduction in viability of primary cortical neurons treated with G4 for 24 h at 0.1 µM in an apoptosis assay was previously reported and supports this idea [24]. In addition, we demonstrated that substitution of 25% of amine groups by both folate and polyethylene glycol molecules diminishes the cytotoxicity, which would be a consequence of the reduction in total positive charge on the PAMAM surface. These results are also in agreement with previous reports in other cell models, where different surface modifications generated less toxic nanocarriers [19,34]. Comparison of the two functionalized dendrimers revealed that PPEG_25_ had a better biocompatibility performance than PFO_25_ since polyethylene glycol (1900–2200 g/mol) is a larger molecule than folate (441.4 g/mol), which decreases the positive charges not only by their substitution, but also by covering the dendrimer surface due to the folding of its long chain [35].

### 2.3. Membrane Permeability Effects

**Figure 3:**
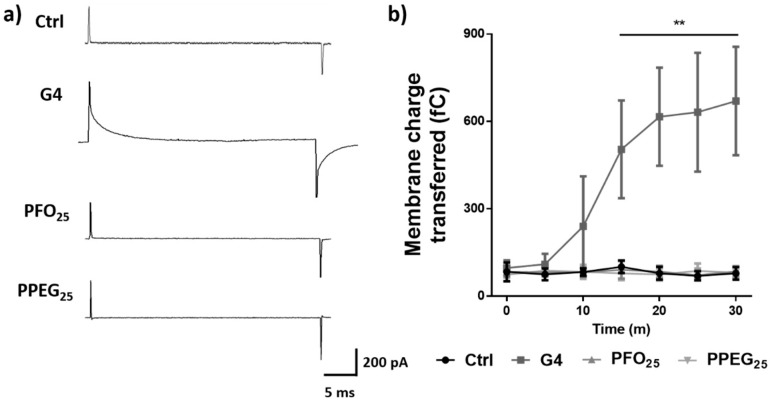
Membrane permeability effects. (**a**) Representative traces of capacitive currents under G4, PFO_25_ and PPEG_25_ treatments at 30 min of recording. Solution without dendrimers was used as control; (**b**) membrane charge transferred for different treatments for 30 min period of recording (*n* = 7, ** *p* < 0.01).

Membrane integrity and ion permeability are important parameters to evaluate in relation to the normal activity of neurons. Previous studies showed associations of the positively charged surface of dendrimers with the alteration of these parameters [12,13]. In order to explore the effects of G4 PAMAM dendrimers on the membrane permeability of hippocampal neurons, cell attached patch clamp recordings were performed. The different dendrimers were included in the internal solution of the recording pipette at 1 µM concentrations, as in perforated patch clamp techniques, and capacitive currents were recorded for 30 min. The amount of current detected through the high resistance seal of the cell attached configuration can be studied in these conditions to evaluate the effect of different substances on neuronal membrane integrity and ion exchange [36,37]. G4 dendrimer treatment showed a significant increase by 7.5-fold in membrane charge transferred of capacitive currents after 15 min of recording, which demonstrates an increment in membrane permeability and loss of membrane integrity. In the case of PFO_25_ and PPEG_25_, significant changes were not observed in capacitive currents during the time of evaluation, indicating that these chemical modifications are able to prevent the effects of G4 dendrimers on membrane permeability (Figure 3).

In regards to other related studies, a previous report has postulated the formation of a Na^+^ selective ion channel by G5 PAMAM dendrimer and the consequent increase of the influx of this ion in pyramidal neurons [31]. An interesting study indicates that G2 and G3 but not G5 dendrimers permeate through α-hemolysin pores and this incorporation of the dendrimers in the lumen of the pore reduces the ionic currents in single-channel recordings [38]. Taken together, our results and the previous reports demonstrate that PAMAM dendrimers can interact with cell membrane components modifying its permeability to ions and the particular effects are dependent on the size and surface properties of these polymers.

### 2.4. Analysis of Intracellular Ca^2+^ Transients

**Figure 4:**
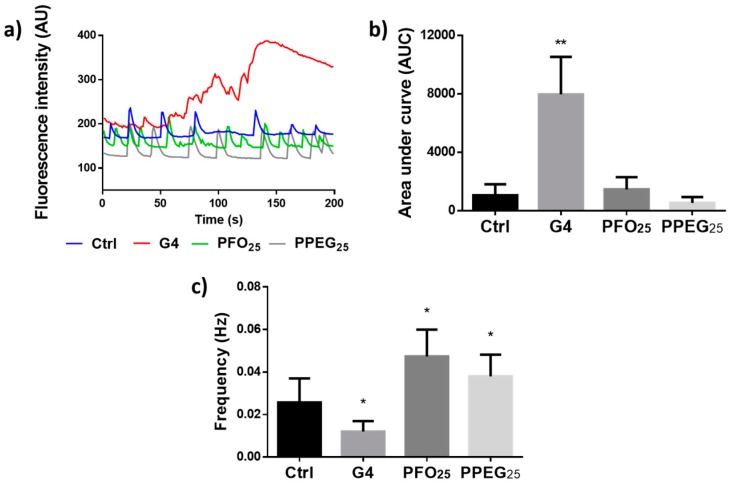
Analysis of intracellular Ca^2+^ transients. (**a**) Representative traces of intracellular Ca^2+^ transients patterns under G4, PFO_25_ and PPEG_25_ treatments. Solution without dendrimers was used as control; (**b**) quantification of the area under the curve for Ca^2+^ transients for different treatments; (**c**) quantification of frequency of Ca^2+^ transients for different treatments (*n* = 15, * *p* < 0.05, ** *p* < 0.01).

The alteration in plasma membrane permeability evidenced in patch clamp recordings could consequently induce the disturbance of homeostatic levels of important ions like Ca^2+^. It has been reported that G5 PAMAM dendrimers increase intracellular Ca^2+^ levels in pyramidal neurons, which determines the disruption of mitochondrial function [32]. To evaluate the possible alterations induced by the dendrimers of our interest on physiological Ca^2+^ activity, intracellular Ca^2+^ transients were measured in the presence of 1 µM solution of the different dendrimers. It was observed that G4 treatment increased intracellular Ca^2+^ with a complete disruption of its normal pattern of transient increments. On the other hand, both PFO_25_ and PPEG_25_ treatments did not disturb the normal pattern of transients (Figure 4a). The intracellular Ca^2+^ increase induced by G4 was not observed when the treatment was performed in external solution without Ca^2+^, supporting the idea that this alteration is due to membrane permeability disruption that allows the massive entrance of extracellular Ca^2+^ (Supporting information—Figure S2). The quantification of the area under curve of the Ca^2+^ transients showed an increase by 8-fold for G4 treatment compared with control condition, demonstrating a significant increment in intracellular Ca^2+^, whereas PFO_25_ and PPEG_25_ did not induce any significant change on this parameter (Figure 4b). The frequency of Ca^2+^ transients was also evaluated, but considering the severe loss of their normal pattern induced by G4 treatment, this result could not be analyzed for this dendrimer. In the case of PFO_25_ and PPEG_25_, a significant increment in Ca^2+^ transients was observed reaching values of 0.047 ± 0.013 and 0.038 ± 0.010 Hz, respectively (Figure 4c). However, this frequency increase would not generate a significant increment in intracellular Ca^2+^ taking into account the results shown in Figure 4b.

### 2.5. Synaptic Vesicle Release Study

**Figure 5:**
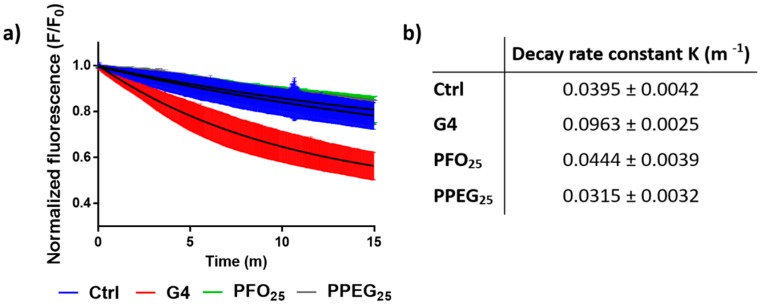
Synaptic vesicle release study. (**a**) Fluorescence intensity decay of FM 1-43 dye under G4, PFO_25_ and PPEG_25_ treatments. Solution without dendrimers was used as control. Black line in curves shows the nonlinear fit regression for each condition; (**b**) decay rate constants *K* of nonlinear fit regression for different treatments.

Considering the role of Ca^2+^ in synaptic vesicle release, we explored if the alterations in Ca^2+^ physiology induced by dendrimers are related with effects on this phenomenon. For the study of synaptic vesicle dynamics in the presence of the dendrimers, fluorescence analysis using the FM 1-43 probe was performed. This amphipathic molecule is capable of marking these vesicles and the decrease in its fluorescent signal intensity is interpreted as a consequence of synaptic vesicle exocytosis [39]. Thus, the fluorescence decay was evaluated in neurons previously stained with the probe under treatment with different dendrimers at 1 µM for 15 min. Results showed that the fluorescence decay kinetics were similar to control conditions when neurons were treated with PFO_25_ and PPEG_25_. Nonetheless, G4 treatment showed a higher decay compared to normal external solution control (Figure 5a). The decay (*K*) constant for the different curves was determined obtaining values of 0.0395 ± 0.0042 min^−1^ for control, 0.0963 ± 0.0025 min^−1^ for G4, 0.0444 ± 0.0039 min^−1^ for PFO_25_, and 0.0315 ± 0.0032 min^−1^ for PPEG_25_ (Figure 5b). The *K* constant for G4 treatment was 2.4-fold higher than the *K* constant for control conditions, whereas similar values to control were obtained for PFO_25_ and PPEG_25_ decay constants. These results demonstrate that only the intracellular Ca^2+^ increment induced by G4 leads to an increase in synaptic vesicle release, and the increments in Ca^2+^ transients frequency induced by PFO_25_ and PPEG_25_ are not correlated with changes in normal synaptic vesicle dynamics.

### 2.6. Effects on Synaptic Activity

**Figure 6:**
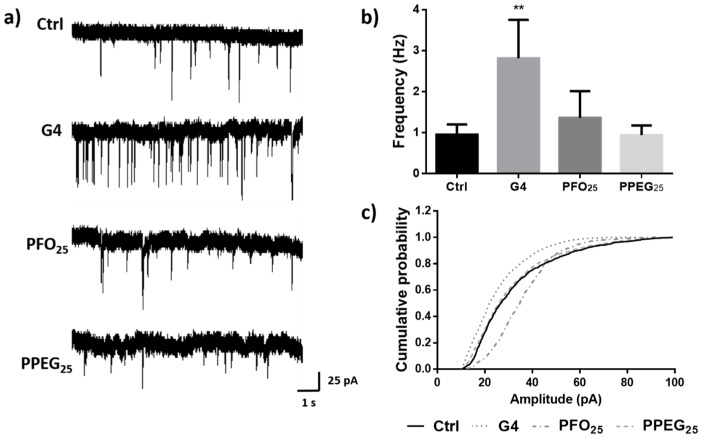
Effects of dendrimer treatments on synaptic activity. (**a**) Representative traces of synaptic activity recordings under G4, PFO_25_ and PPEG_25_ treatments. Solution without dendrimers was used as control; (**b**) quantification of synaptic activity frequency for different treatments (*n* = 9, ** *p* < 0.01); (**c**) cumulative probability of amplitude values for different treatments. No significant differences were found.

After the synaptic vesicle release experiments, it was of interest to study the effect of the different dendrimers on spontaneous synaptic activity. Therefore, whole cell patch clamp recordings were performed applying the different dendrimers at 1 µM by extracellular perfusion (Figure 6a). Results showed a significant increase in the frequency of synaptic events when neurons were treated with G4 (2.81 ± 0.93 Hz), which was around 3-fold higher compared with control (0.95 ± 0.25 Hz). However, no significant differences were observed for PFO_25_ and PPEG_25_ treatments (Figure 6b). Amplitude was also evaluated, but no significant differences were determined for any treatment (Figure 6c).

In terms of neuronal function alterations, the results of this study demonstrate that the membrane permeability disturbance induced by G4 is able to generate a marked increase of Ca^2+^ into the neurons, which leads to an increment in synaptic vesicle release and a consequent increase in the frequency of synaptic activity. These results demonstrate that positively charged G4 dendrimers induce a significant increment in presynaptic excitability of hippocampal neurons, disrupting their normal synaptic transmission. Future experiments should be directed at determining the participation of specific molecular mechanisms in this phenomenon.

Interestingly, the substitution of 25% of dendrimer terminal groups with folate and polyethylene glycol is sufficient to prevent the membrane permeability alteration, intracellular Ca^2+^ dysregulation, increment in synaptic vesicle release and synaptic activity frequency, as shown with the results obtained with PFO_25_ and PPEG_25_ dendrimers.

## 3. Materials and Methods

### 3.1. Materials

Fourth generation PAMAM dendrimer (G4), polyethylene glycol 2000 and folic acid were purchased from Sigma-Aldrich (Santiago, Chile). Fluo-4, AM, cell permeant probe, FMTM1-43 dye (*N*-(3-Triethylammoniumpropyl)-4-(4-(Dibutylamino) Styryl) Pyridinium Dibromide) and AlamarBlueTM cell viability reagents were purchased from Thermo Fischer Scientific (Santiago, Chile).

### 3.2. Cell Cultures

Hippocampal neurons were obtained from 18–19 days C57BL/J6 mice embryos as previously described in accordance with National Institutes of Health (NIH) recommendations [40]. The neuronal feeding medium consisted of 90% minimal essential medium (MEM, BRL Technologies, Rockville, MD, USA), 5% heat-inactivated horse serum, 5% fetal bovine serum and a mixture of nutrient supplements. Cells were plated at 300,000 cells/mL and studied after 7–10 days in culture. Care of animals and the experimental protocols of this study were approved by the Institutional Animal Use Committee of the University of Concepción and conducted according to the ethical protocols established by the NIH and the National Committee of Science and Technology (CONICYT).

### 3.3. Functionalization of PAMAM Dendrimers

Three different PAMAM dendrimers were used: (1) unmodified G4 PAMAM dendrimer that has 64 positively charged amine surface groups exposed (G4), (2) G4 PAMAM with 25% of its amine surface groups modified with folate (PFO_25_) following the protocol of Benchaala et al. [41], and (3) G4 PAMAM with 25% of its amine surface groups modified with polyethylene glycol (PPEG_25_) following the protocol of Kojima et al. [42]. Dialysis and washes were applied to the dendrimers. After a drying process, dendrimers were dissolved in PBS buffer (pH 7.4). Chemical modifications and absence of methanol and other reagents were verified by ^1^H-NMR spectroscopy in a Bruker 400 Avance spectrometer (Ettlingen, Germany) (Supporting information—Figure S1).

### 3.4. Cytotoxicity Evaluation

Hippocampal neurons were incubated in 24-well plates for 24 h at 37 °C and 5% CO_2_ with different dendrimers at 0.1, 1, 10 and 100 µM. Neurons incubated only with culture media and with 0.25% Triton were used as positive and negative controls of viability, respectively. After treatment, neurons were incubated 2 h with 10% AlamarBlue reagent at 37 °C and 5% CO_2_. Finally, reagent fluorescence (excitation 540 nm–emission 590 nm) was measured in a NOVOstar multiplate reader (BGM Labtech, Offenburg, Germany).

### 3.5. Analysis of Intracellular Ca^2+^ Transients

Hippocampal neurons were incubated with 5 µM Fluo4-AM probe for 20 min in PBS buffer at 37 °C and 5% CO_2_. Subsequently, cells were washed 20 min with PBS and plated in normal external solution consisting of 150 mM NaCl, 5.4 mM KCl, 2.0 mM CaCl_2_, 1.0 mM MgCl_2_, 10 mM HEPES (pH 7.4) and 10 mM glucose. Then, cells were mounted on a Nikon TE2000 inverted microscope (Tokyo, Japan). Changes in fluorescence (excitation 480 nm–emission 510 nm) were acquired every 2 s over a period of 200 s with an iXon EMCCD camera (Andor, Belfast, Ireland). The recordings were performed with ImagingWorkbench 6.0 (Indec Biosystems, Santa Clara, CA, USA). Recordings in the presence of normal external solution were used as control and then 1 µM solution of different dendrimers was added to evaluate the dendrimer treatment effect.

### 3.6. Electrophysiology

All patch clamp recordings were performed using a holding potential of −60 mV and currents were acquired with Digidata 1200 board and the pClamp10 software (Axon Instruments, Inc., Foster City, CA, USA). Recording pipettes were pulled from borosilicate glass (WPI, Sarasota, FL, USA) on a horizontal puller (Sutter Instruments, Novato, CA, USA). Patch electrodes were filled with 140 mM KCl, 10 mM BAPTA, 10 mM HEPES (pH 7.4), 4 mM MgCl_2_, 2 mM ATP and 0.5 mM GTP. Normal external solution for cells was the same as described in the analysis of intracellular Ca^2+^ transients protocol. For the study of membrane permeability, cell attached configuration was performed adding the different dendrimers in the pipette solution at 1 µM as in perforated patch clamp techniques and solution without dendrimers was used as control. Recordings were performed for 30 min. For synaptic activity evaluation, whole cell configuration was used. Normal external solution was applied by perfusion for two minutes as control and then different dendrimers in normal external solution at 1 µM were applied under the same conditions. In addition, 0.5 µM Tetrodotoxin (TTX) was used to block action potentials.

### 3.7. Synaptic Vesicle Release Study

Hippocampal neurons were incubated in high K^+^ solution (30 mM) for 5 min. Then, cells were washed and incubated with 15 µM FM 1-43 dye for 15 min for synaptic vesicles labeling. Neurons were washed again and mounted on a Nikon TE2000 inverted microscope. Changes in fluorescence (excitation 510 nm–emission 620 nm) were acquired every 2 s over a period of 15 min with an iXon EMCCD camera. The recordings were made with ImagingWorkbench 6.0. Recordings in the presence of 1 µM solution of the different dendrimers were performed and the same normal external solution described in the analysis of intracellular Ca^2+^ transients protocol was used as the control.

### 3.8. Statistical Analyses

All experiments were performed using for convenience nonprobability sampling. Each primary culture neuron from three different dissections was the study unit. For cytotoxicity evaluation, each well of 24-well plates of primary cultures from three different dissections was considered as the study unit. The Shapiro–Wilk test was used for the evaluation of the normality of the variables. Analyses of variances (ANOVA) and Tukey’s post hoc tests were used to determine significant differences. In the case of the evaluation of synaptic activity amplitude, the Kolmogorov-Smirnov test was performed. All data analysis was made with GraphPad Prism 6.0 software (GraphPad, La Jolla, CA, USA).

## 4. Conclusions

Positively charged G4 PAMAM dendrimers induce important toxic effects in hippocampal neurons that lead to a significant reduction in viability. G4 treatment generates a disruption in the normal permeability of the neuron membrane, which conduces to a massive Ca^2+^ intake. This augmentation in intracellular Ca^2+^ increases the synaptic vesicle release and frequency of synaptic events. The substitution of 25% of amine surface groups of dendrimers with folate and polyethylene glycol is able to prevent this synaptic dysregulation and decrease in cell viability. These results validate the hypothesis that unmodified dendrimers generate neurotoxic effects and alter the normal synapsis, highlighting the adverse consequences of using non-functionalized PAMAM dendrimers. Surface functionalization of PAMAM dendrimers, on the other hand, can prevent these adverse effects, opening the possibility for generating biocompatible nanocarriers for safe CNS therapeutic applications.

## Supplementary Materials

The following are available online at www.mdpi.com/2079-4991/8/1/7/s1, Figure S1: ^1^H-NMR spectres for modified dendrimers; Figure S2: Analysis of intracellular Ca^2+^ transients without extracellular Ca^2+^.

## References

[1] Jain N.K., Gupta U. (2008). Application of dendrimer—Drug complexation in the enhancement of drug solubility and bioavailability. Expert Opin. Drug Metab. Toxicol..

[2] Pérez-Martínez F.C., Guerra J., Posadas I., Ceña V. (2011). Barriers to non-viral vector-mediated gene delivery in the nervous system. Pharm. Res..

[3] Wu L.P., Ficker M., Christensen J.B., Trohopoulos P.N., Moghimi S.M. (2015). Dendrimers in Medicine: Therapeutic Concepts and Pharmaceutical Challenges. Bioconjug. Chem..

[4] Lee C.C., MacKay J.A., Fréchet J.M.J., Szoka F.C. (2005). Designing dendrimers for biological applications. Nat. Biotechnol..

[5] Kannan R.M., Nance E., Kannan S., Tomalia D.A. (2014). Emerging concepts in dendrimer-based nanomedicine: From design principles to clinical applications. J. Intern. Med..

[6] Cheng C.J., Tietjen G.T., Saucier-Sawyer J.K., Saltzman W.M. (2015). A holistic approach to targeting disease with polymeric nanoparticles. Nat. Rev. Drug Discov..

[7] Manchun S., Dass C.R., Sriamornsak P. (2012). Targeted therapy for cancer using pH-responsive nanocarrier systems. Life Sci..

[8] Wu J., Huang W., He Z. (2013). Dendrimers as carriers for siRNA delivery and gene silencing: A review. Sci. World J..

[9] Kesharwani P., Jain K., Jain N.K. (2014). Dendrimer as nanocarrier for drug delivery. Prog. Polym. Sci..

[10] Duncan R., Izzo L. (2005). Dendrimer biocompatibility and toxicity. Adv. Drug Deliv. Rev..

[11] Jain K., Kesharwani P., Gupta U., Jain N.K. (2010). Dendrimer toxicity: Let’s meet the challenge. Int. J. Pharm..

[12] Hong S., Bielinska A.U., Mecke A., Keszler B., Beals J.L., Shi X., Balogh L., Orr B.G., Baker J.R., Banaszak Holl M.M. (2004). Interaction of poly(amidoamine) dendrimers with supported lipid bilayers and cells: Hole formation and the relation to transport. Bioconjug. Chem..

[13] Leroueil P.R., Berry S.A., Duthie K., Han G., Rotello V.M., Mcnerny D.Q., Baker J.R., Orr B.G., Holl M.M.B. (2008). Wide Varieties of Cationic Nanoparticles Induce Defects in Supported Lipid Bilayers. Nano Lett..

[14] Janaszewska A., Ciolkowski M., Wróbel D., Petersen J.F., Ficker M., Christensen J.B., Bryszewska M., Klajnert B. (2013). Modified PAMAM dendrimer with 4-carbomethoxypyrrolidone surface groups reveals negligible toxicity against three rodent cell-lines. Nanomed. Nanotechnol. Biol. Med..

[15] Mukherjee S.P., Byrne H.J. (2013). Polyamidoamine dendrimer nanoparticle cytotoxicity, oxidative stress, caspase activation and inflammatory response: Experimental observation and numerical simulation. Nanomedicine.

[16] Wang W., Xiong W., Wan J., Sun X., Xu H., Yang X. (2009). The decrease of PAMAM dendrimer-induced cytotoxicity by PEGylation via attenuation of oxidative stress. Nanotechnology.

[17] Enciso A.E., Neun B., Rodriguez J., Ranjan A.P., Dobrovolskaia M.A., Simanek E.E. (2016). Nanoparticle effects on human platelets in vitro: A comparison between PAMAM and triazine dendrimers. Molecules.

[18] Jevprasesphant R., Penny J., Jalal R., Attwood D., Mckeown N.B., Emanuele A.D. (2003). The influence of surface modification on the cytotoxicity of PAMAM dendrimers. Int. J. Pharm..

[19] Ciolkowski M., Petersen J.F., Ficker M., Janaszewska A., Christensen J.B., Klajnert B., Bryszewska M. (2012). Surface modification of PAMAM dendrimer improves its biocompatibility. Nanomed. Nanotechnol. Biol. Med..

[20] Del Burgo L.S., Hernández R.M., Orive G., Pedraz J.L. (2014). Nanotherapeutic approaches for brain cancer management. Nanomed. Nanotechnol. Biol. Med..

[21] Xu L., Zhang H., Wu Y. (2014). Dendrimer advances for the central nervous system delivery of therapeutics. ACS Chem. Neurosci..

[22] Saraiva C., Praça C., Ferreira R., Santos T., Ferreira L., Bernardino L. (2016). Nanoparticle-mediated brain drug delivery: Overcoming blood—Brain barrier to treat neurodegenerative diseases. J. Control. Release.

[23] Cerqueira S.R., Silva B.L., Oliveira J.M., Mano J.F., Sousa N., Salgado A.J., Reis R.L. (2012). Multifunctionalized CMCht/PAMAM Dendrimer Nanoparticles Modulate the Cellular Uptake by Astrocytes and Oligodendrocytes in Primary Cultures of Glial Cells. Macromol. Biosci..

[24] Albertazzi L., Gherardini L., Brondi M., Sato S.S., Bifone A., Pizzorusso T., Ratto G.M., Bardi G. (2013). In Vivo Distribution and Toxicity of PAMAM Dendrimers in the Central Nervous System Depend on Their Surface Chemistry. Mol. Pharm..

[25] Vidal F., Vásquez P., Díaz C., Nova D., Alderete J., Guzmán L. (2016). Mechanism of PAMAM Dendrimers Internalization in Hippocampal Neurons. Mol. Pharm..

[26] Kannan S., Dai H., Navath R.S., Balakrishnan B., Jyoti A., Janisse J., Romero R., Kannan R.M. (2012). Dendrimer-Based Postnatal Therapy for Neuroinflammation and Cerebral Palsy in a Rabbit Model. Sci. Transl. Med..

[27] Huang R., Ma H., Guo Y., Liu S., Kuang Y., Shao K., Li J., Liu Y., Han L., Huang S. (2013). Angiopep-conjugated nanoparticles for targeted long-term gene therapy of parkinson’s disease. Pharm. Res..

[28] Liu S., Guo Y., Huang R., Li J., Huang S., Kuang Y., Han L., Jiang C. (2012). Gene and doxorubicin co-delivery system for targeting therapy of glioma. Biomaterials.

[29] Vidal F., Guzman L. (2015). Dendrimer nanocarriers drug action: Perspective for neuronal pharmacology. Neural Regen. Res..

[30] Wang S., Li Y., Fan J., Wang Z., Zeng X., Sun Y., Song P., Ju D. (2014). The role of autophagy in the neurotoxicity of cationic PAMAM dendrimers. Biomaterials.

[31] Nyitrai G., Keszthelyi T., Bota A., Simon A., Toke O., Horvath G., Pal I., Kardos J., Heja L. (2013). Sodium selective ion channel formation in living cell membranes by polyamidoamine dendrimer. Biochim. Biophys. Acta.

[32] Nyitrai G., Héja L., Jablonkai I., Pál I., Visy J., Kardos J. (2013). Polyamidoamine dendrimer impairs mitochondrial oxidation in brain tissue. J. Nanobiotechnol..

[33] Wicki A., Witzigmann D., Balasubramanian V., Huwyler J. (2015). Nanomedicine in cancer therapy: Challenges, opportunities, and clinical applications. J. Control. Release.

[34] Janaszewska A., Studzian M., Petersen J.F., Ficker M., Christensen J.B., Klajnert-Maculewicz B. (2015). PAMAM dendrimer with 4-carbomethoxypyrrolidone-In vitro assessment of neurotoxicity. Nanomed. Nanotechnol. Biol. Med..

[35] Barraza L.F., Jiménez V.A., Alderete J.B. (2017). Association of Methotrexate with Native and PEGylated PAMAM-G4 Dendrimers: Effect of the PEGylation Degree on the Drug-Loading Capacity and Preferential Binding Sites. J. Phys. Chem. B.

[36] Sepulveda F.J., Parodi J., Peoples R.W., Opazo C., Aguayo L.G. (2010). Synaptotoxicity of Alzheimer beta amyloid can be explained by its membrane perforating property. PLoS ONE.

[37] Peters C., Espinoza M.P., Gallegos S., Opazo C., Aguayo L.G. (2015). Alzheimer’s Aβ interacts with cellular prion protein inducing neuronal membrane damage and synaptotoxicity. Neurobiol. Aging.

[38] Martin H., Kinns H., Mitchell N., Astier Y., Madathil R., Howorka S. (2007). Nanoscale Protein Pores Modified with PAMAM Dendrimers. J. Am. Chem. Soc..

[39] Gaffield M.A., Betz W.J. (2006). Imaging synaptic vesicle exocytosis and endocytosis with FM dyes. Nat. Protoc..

[40] Aguayo L.G., Pancetti F.C. (1994). Ethanol modulation of the gamma-aminobutyric acidA- and glycine-activated Cl- current in cultured mouse neurons. J. Pharmacol. Exp. Ther..

[41] Benchaala I., Mishra M.K., Wykes S.M., Hali M., Kannan R.M., Whittum-Hudson J.A. (2014). Folate-functionalized dendrimers for targeting Chlamydia-infected tissues in a mouse model of reactive arthritis. Int. J. Pharm..

[42] Kojima C., Kono K., Maruyama K., Takagishi T. (2000). Synthesis of polyamidoamine dendrimers having poly(ethylene glycol) grafts and their ability to encapsulate anticancer drugs. Bioconjug. Chem..

