# Supplementary material for: Prevention of Synaptic Alterations and Neurotoxic Effects of PAMAM Dendrimers by Surface Functionalization
Source: Nanomaterials (Basel). 2017 Dec 25;8(1):7. doi: 10.3390/nano8010007 (PMC5791094; doi:10.3390/nano8010007)
Supplement: Supplementary file 1 [file nanomaterials-08-00007-s001.pdf]

nanomaterials

Supplementary Materials

Prevention of Synaptic Alterations and Neurotoxic Effects of PAMAM Dendrimers by Surface Functionalization

Felipe Vidal [1], Pilar Vásquez [1], Francisca R. Cayumán [1], Carola Díaz [2], Jorge Fuentealba [3], Luis G. Aguayo [3], Gonzalo E. Yévenes [3], Joel Alderete [2] and Leonardo Guzmán [1,*]

1. Laboratory of Molecular Neurobiology, Department of Physiology, Faculty of Biological Sciences, University of Concepcion, Concepción 4070386, Chile; felipevidal@udec.cl (F.V.); pivasquez@udec.cl (P.V.); fcayuman@udec.cl (F.R.C.)
2. Laboratory of Biomaterials and Molecular Design, Department of Organic Chemistry, Faculty of Chemical Sciences, University of Concepcion, Concepción 4070386, Chile; caroladiaz@udec.cl (C.D.); jalderet@udec.cl (J.A.)
3. Department of Physiology, Faculty of Biological Sciences, University of Concepcion, Concepción 4070386, Chile; jorgefuentealba@udec.cl (J.F.); laguayo@udec.cl (L.G.A.); gyevenes@udec.cl (G.E.Y.)

* Correspondence: joseguzman@udec.cl; Tel.: 56-412661229; Fax: 56-412245975

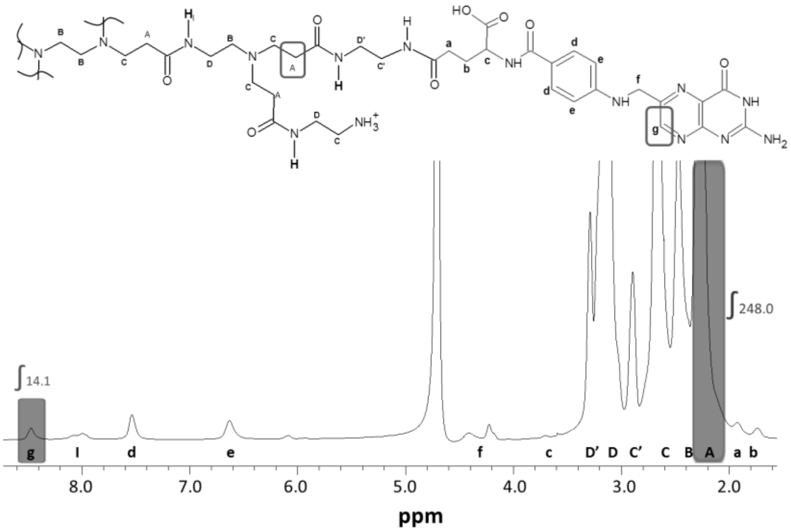

H	δ (ppm)	Integral
b	1.74	-
a	1.93	-
A	2.22	248.0*
B	2.47	-
C′	2.68	-
C	2.89	-
D′	3.17	-
D	3.29	-
c	3.60	-
f	4.42	30.8
e	6.63	29.8
d	7.54	31.6
I	8.05	-
g	8.47	14.1

(**a**)

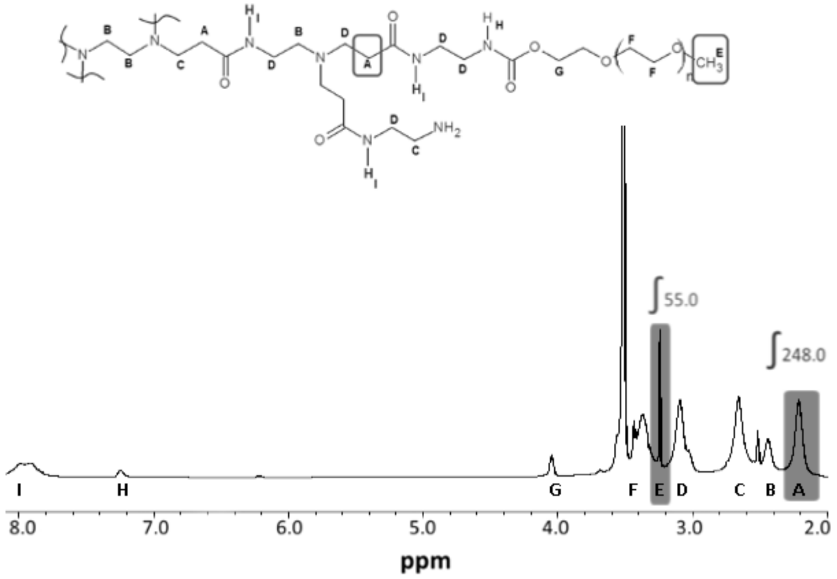

H	δ (ppm)	Integral
A	2.21	248.0*
B	2.48	108.7
C	2.66	302.5
D	3.09	313.7
E	3.24	55.0
F	3.44	1169
G	4.04	33.3
H	7.24	10.3
I	7.87	116.8

(**b**)

Figure S1. ^{1}H NMR spectra for modified dendrimers. (**a**) ^{1}H NMR (D_2O, 400 MHz) spectra for PFO_{25} dendrimer; (**b**) ^{1}H NMR (DMSO-d6, 400 MHz) spectra for $PPEG_{25}$ dendrimer.

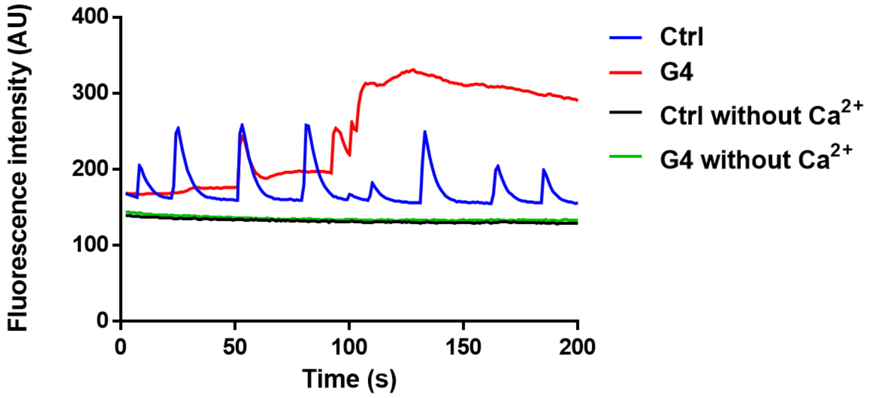

Figure S2. Analysis of intracellular Ca^{2+} transients without extracellular Ca^{2+}. In order to corroborate that intracellular Ca^{2+} increment induced by G4 is due to the intake of extracellular Ca^{2+} and not because of the release of organelles storage, G4 treatment was performed using an external solution without Ca^{2+}. No changes in intracellular Ca^{2+} are observed in this condition; (n = 10).

© 2017 by the authors. Submitted for possible open access publication under the terms and conditions of the Creative Commons Attribution (CC BY) license (http://creativecommons.org/licenses/by/4.0/).
